# Characterization of In Vivo Function(s) of Members of the Plant Mitochondrial Carrier Family

**DOI:** 10.3390/biom10091226

**Published:** 2020-08-24

**Authors:** Adriano Nunes-Nesi, João Henrique F. Cavalcanti, Alisdair R. Fernie

**Affiliations:** 1 Departamento de Biologia Vegetal, Universidade Federal de Viçosa, Viçosa 36570-900, Minas Gerais, Brazil; 2 Instituto de Educação, Agricultura e Ambiente, Universidade Federal do Amazonas, Humaitá 69800-000, Amazonas, Brazil; jcavalcanti@ufam.edu.br; 3 Max-Planck-Instiute of Molecular Plant Physiology, 14476 Postdam-Golm, Germany

**Keywords:** mitochondrial carrier family, MCF, function, mitochondria, plant metabolism, plant development

## Abstract

Although structurally related, mitochondrial carrier family (MCF) proteins catalyze the specific transport of a range of diverse substrates including nucleotides, amino acids, dicarboxylates, tricarboxylates, cofactors, vitamins, phosphate and H^+^. Despite their name, they do not, however, always localize to the mitochondria, with plasma membrane, peroxisomal, chloroplast and thylakoid and endoplasmic reticulum localizations also being reported. The existence of plastid-specific MCF proteins is suggestive that the evolution of these proteins occurred after the separation of the green lineage. That said, plant-specific MCF proteins are not all plastid-localized, with members also situated at the endoplasmic reticulum and plasma membrane. While by no means yet comprehensive, the in vivo function of a wide range of these transporters is carried out here, and we discuss the employment of genetic variants of the MCF as a means to provide insight into their in vivo function complementary to that obtained from studies following their reconstitution into liposomes.

## 1. Introduction

The characterization of heterologously expressed, liposome-reconstituted proteins has provided considerable insight into the transport capacities of human, yeast and plant MCF members [1,2,3,4,5,6,7,8,9,10,11,12,13]. Moreover, inference from phylogenetic analysis has been demonstrated, at least in some cases, to provide good hints as to the function of plant MCF proteins on the basis of the experimentally characterized functions of, for example, yeast proteins with which they share high homology [14]. While these studies have provided a wealth of information concerning the potential substrates that can be transported by a relatively large number of the transporters [15], the biological relevance of these properties remains dependent on the context of where and when they are expressed. The advent of next-generation technologies, and before that of microarrays, has led to the establishment of considerable data regarding the expression of MCF members in Arabidopsis [16,17,18,19] and several other species ([20,21]). Bioinformatic analysis has revealed that, as for the previous identification of an Arabidopsis monolignol transporter [22], co-expression analysis provides clues as to the in vivo function of a range of MCF transporters [23]. In addition, comparative studies are naturally confined to the subfamilies which are conserved across the eukaryotic lineage [14]. Succinate/fumarate carriers are apparently absent in animals while yeasts appear to lack uncoupling proteins (UCPs). The presence of a subfamily in two of the three lineages suggests that it is likely to have been lost—perhaps due to compensation by other transporters rendering it redundant [24]. By contrast, the presence of a subfamily in a single lineage, for example the mitochondrial GTP/GDP transporter of yeast or the plastidial adenine nucleotide carriers and Brittle1 protein, is likely indicative of an innovation that occurred after the separation of the eukaryotes. In certain functional clades, plants exhibit a higher number of paralogs (although many of these remain to be experimentally proven), which may indicate that gene duplication and/ or retention occurred more often for these transporters in plants. Indeed, this is highly likely, given the documented fact that gene duplication is generally considerably more prominent in plants [25]. While it is tempting to suggest that the increased number of transporters gives flexibility to immotile plants [24], this is unlikely to be the reason, since mobile algae also possess, at least for some clades, more MCFs than human or yeast [14]. A more attractive proposition is thus that plants contain more MCF proteins due to the presence of the plastid organelle [24]. While arguments for this were initially made on comparison of the NAD transporters NDT1 and NDT2 [24], with the former thought to be a plastidial and the latter a mitochondrial localized transporter [26]; this needs revision now that, as detailed below, both have been reclassified as mitochondrial [27,28]. That said, there are several proteins that exhibit dual targeting and thus may fulfill different functions at the different locations. Examples include the dual mitochondrial and plastidial localized SAM [29,30,31] and Brittle1 transporters [32,33,34]. Moreover, it is important to note that plant specific MCF proteins including the adenine nucleotide carriers ER-ANT1 and PM-ANT1 do not only comprise plastidial carriers [35,36]. In this article, we will summarize functional insights obtained from evaluating spatio-temporal differences in expression, subcellular localization and finally from the study of transgenic plants exhibiting altered expression of the transporter(s) of interest.

## 2. Expression of MCF Members

### 2.1. Environmental Specific Gene Expression

**Figure 1:**
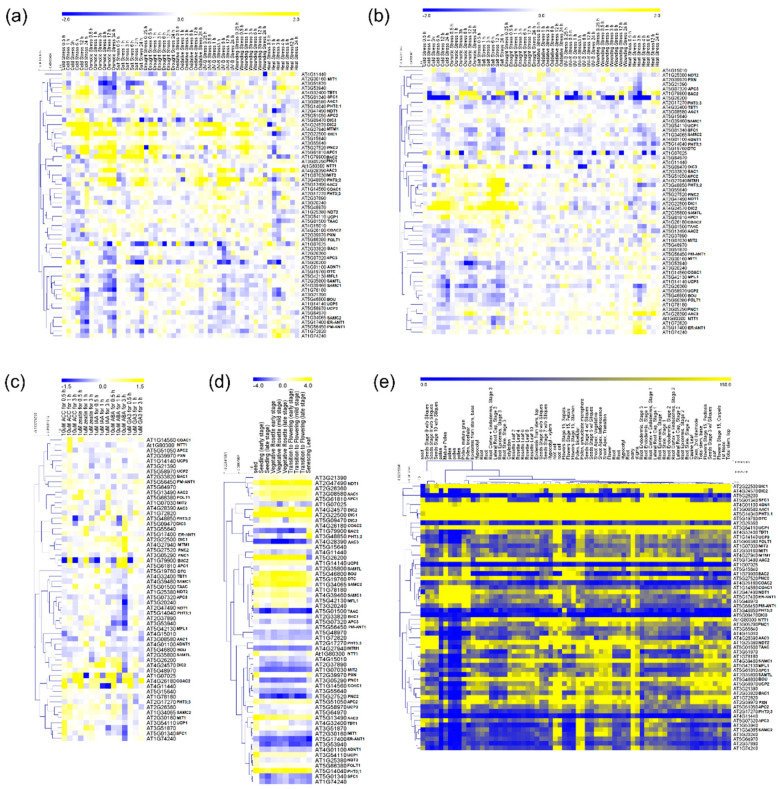
Hierarchical cluster of gene expression analysis of mitochondrial carrier family (MCF) genes of *Arabidopsis thaliana*. Heat map of MCF genes in shoots (**a**) and roots (**b**) of plants under a range of stress situations. Heat map of MCF genes expression in plants submitted to hormone treatment (**c**), throughout plant development (**d**) and in several tissues (**e**). The values are stated as log2 ratio (**a**–**d**) and relative value (**e**). The complete data set is presented in the supplemental information online (Supplementary File S1). For definitions of gene names, please see the main text.

To have a broad view of the expression profile exhibited by MCF genes we used information available in The Bio-Analytical Resource for Plant Biology database [37,38] and prepared a heatmap documenting the relative expression levels of 58 MCs in *A. thaliana* within a wide range of tissues and environmental conditions (Figure 1). We first turned our attention to the MCF gene expression levels under a range of stresses concerning exposure to cold, osmotic, salt, drought, oxidative, UV-B, wounding and heat stress in shoots and roots (Figure 1a,b). Interestingly, out of 58 MCFs, eight transporters (BAC2, DIC2, MTM1, DIC1, PNC2, APC1, PHT3;2 and AAC2) display expression profiles that are highly upregulated in shoots under conditions of cold, osmotic and salt stress whereas the other 50 genes were generally characterized as displaying lower and more specific changes (Figure 1a). In general, in root tissues the expression profile of plants under different stress conditions appears to be independent of the alterations verified in shoots suggesting a molecular plasticity between the tissues in response to environmental stress (Figure 1a,b). However, seven transporters (BAC2, DIC1, DIC2, MTM1, PHT3;2, AT3G55640 and PNC2) are highly upregulated under cold, osmotic and salt stress (Figure 1b). Of the seven genes highly expressed in roots, six were also upregulated in shoots, but the importance of these genes seems to be different during the stress in the two organs. For example, BAC2, an amino acid transporter, exhibits a moderate expression in roots of plants submitted to few hours of osmotic and salt stress (Figure 1b); however in shoots BAC2 is highly upregulated shortly after the beginning of stress application, remaining highly expressed during the whole period of stress exposure (Figure 1a). In this context, it is noteworthy that compelling evidence suggests that BAC2 plays a role in mechanisms of nitrogen recycling during stress establishment and recovery [39,40]. Furthermore, genes encoding UCP subfamily members are highlighted as stress-response genes [41,42]. Even though their role in plant metabolism is currently unclear, evidence suggests that UCP activity acts to dissipating the proton gradient generated on ATP synthesis while preventing the accumulation of reactive oxygen species under stress [43]. However, UCP isoforms are specifically induced and/or repressed in diverse conditions, despite being generally downregulated (Figure 1a). The transcript levels of UCP1 and UCP2 were not induced under the different stress conditions. This finding is in agreement with a study by Van Aken et al. [44], which suggests that the UCP proteins are not among the most stress responsive mitochondrial proteins. Other interesting patterns are also apparent from our in silico expression analysis; for example, when the expression profile of PiC2 (a phosphate transporter also known as PHT3:2) is studied, distinct patterns are apparent in roots and shoots (Figure 1a,b). In roots only, a moderate increase in expression was observed after 6-h exposure to salt stress, while in shoots, gene expression was highly upregulated by cold, osmotic, salt, oxidative, UV-B and wounding stresses. Differential root and shoot expression patterns are also apparent for DIC1, DIC2 and PNC2 displaying differences in expression under cold and salt stress in roots and cold, osmotic, salt, oxidative, UV-B and wounding stress in shoots, respectively.

### 2.2. Hormone Treatment Gene Expression

Differential gene expression profiling following hormone treatment provides interesting clues as to the putative roles of MCF members within plant metabolism. Compelling evidence suggests a close association between hormones with energy metabolism [45,46]. However, to date, there is a lack of studies into the role of MCF members in this vein. Our meta-analysis of the Arabidopsis expression profiling database [37,38] reveals that hormone application MCF members are most responsive to abscisic acid (ABA), with seven genes affected negatively by application of ABA (APC3, AT2G37890, AT3G53940, MFL1, AAC1, AT3G51870, SFC1). By contrast, BAC2, APC1 and COAC2 are highly upregulated following ABA treatment (Figure 1c). Interestingly, APC family members exhibit differential dynamics influenced by ABA, with APC1 being upregulated while APC3 decreases following application of ABA. While these observations suggest that shed MCFs may be associated with hormone effects in vivo investigations are required in order to confirm this.

### 2.3. Developmental-Specific Gene Expression

Since transporters are an essential component linking the entirety of cellular metabolism and integrating branched biochemical pathways among subcellular compartmentalization their importance throughout the plant lifespan can be anticipated (Figure 1d). MCF gene expression profiling across development shows two distinct patterns, with one set of genes being upregulated while others are downregulated. Some insight into MCF function may be retrieved from Figure 1d. The expression of adenylate carriers at specific stages of development (Figure 1d) indicates a strong induction of AAC1 and APC1 in senescent leaves. Interestingly, a moderate induction of expression of AAC2 and APC2 is observed in the same tissues, concomitantly with a reduction of AAC3, ADNT1, ER-ANT1 and PM-ANT1 expression. Thus, it appears that natural senescence causes remarkable changes in the expression of different adenylate carriers in plants [23]. Furthermore, BAC2 displays low transcript levels in most development stages, even lower levels during shoot senescence and higher expression in senescing leaves (Figure 1d). Nevertheless, it has been suggested that BAC2 plays a role during senescence being involved in nitrogen remobilization [39,40]. Considering that adenylate carriers might also act during natural senescence [23], it appears that BAC2 shows a synergy with energy generation beyond nitrogen recycling per se. Since natural senescence is characterized by carbohydrate starvation, amino acid degradation (e.g., lysine and arginine; both amino acids transported by BAC2) by mitochondria can most likely sustain energy demand by the cell [47,48,49]. Despite the association between adenylate carriers and BAC2 transporter being interesting, further experimental validation is required to ensure that the observed coexpression is biologically relevant and to test the above-mentioned hypothesis.

### 2.4. Tissue-Specific Gene Expression

We next evaluated the expression profiles of MCF genes across plant tissues (Figure 1e). Several MCF transporters (for example AT4G11440, SFC1) are expressed constitutively in different tissues, suggesting that they are involved in essential housekeeping functions. By contrast, the majority of the biochemically characterized MCFs are differentially expressed among different cell types. Particularly, the NAD transporter NDT1 [27], AAC1, AAC2, and SAMC1 [29,31] are highly expressed in mature pollen and also developing seeds, which is in agreement with the reported import of NAD, ADP and methionine [31,50] into mitochondria of these tissues. Other transporters, including CAC, DIC1, DIC2, NDT1, AAC1, AAC2, BAC2 and a few, as yet, uncharacterized proteins, were predominantly expressed in pollen, seeds and vegetative rosettes, while many genes of unknown function were predominantly expressed in embryo and seedling stages or in heterotrophic root and stem tissues. As for the other transcript data described above, it is important to note that conclusions related to function await validation.

## 3. Subcellular Localization of MCF Members and Characterization of Lines Deficient in the Expression of the Transporters

**Figure 2:**
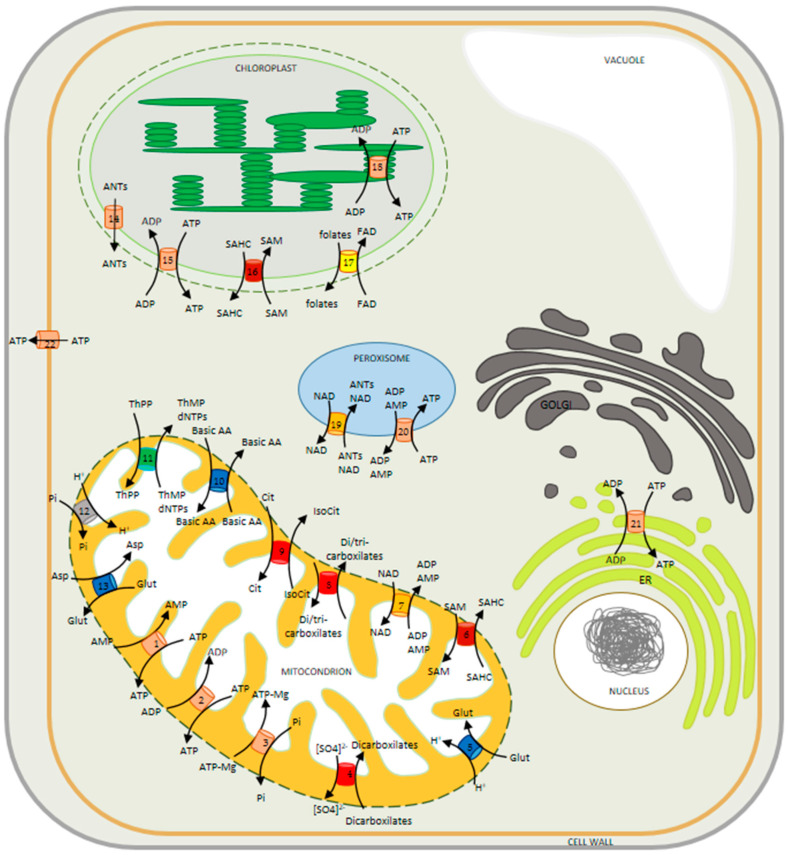
Model illustrating the mitochondrial carriers described and characterized in plant cells. All carriers belong to the mitochondrial carrier family (MCF). Carriers localized in the inner mitochondrial membrane: (1) ATP/AMP carrier, ADNT1; (2) ATP/ADP carriers, AACs; (3) AMP-ADP-ATP/Pi carriers, APCs; (4) dicarboxylate carriers, DIC1-3; (5) glutamate-H^+^ carrier, BOU; (6) *S*-adenosylmethionine carrier, SAMC1; (7) NAD carriers, NDT1-2; (8) dicarboxylate/tricarboxylate carrier, DTC; (9) Citrate/isocitrate carrier, SFC1; (10) basic aminoacids carries BAC1-2; (11) thiamine pyrophosphate carrier; TPC; (12) phosphate (Pi) carriers, PiC1-3; (13) uncoupling protein, UCP1-2. Carriers localized in the inner membrane of chloroplast: (14) ATP/ADP/AMP exporter, ATBT1; (15) ATP/ADP carrier, NTT1; (16) *S*-adenosylmethionine carrier, SAMC1; (17) folates transporter, FOLT1. Carrier localized in the membrane of thylakoid: (18) ATP/ADP transporter, TAAC. Carriers localized in the membranes of peroxisome [NAD transporter (19); adenine nucleotide carriers, PNC1/2 (20)], endoplasmic reticulum [ATP/ADP exchanger, ER-ANT1 (21)] and plasma membrane [ATP exporter, PM-ANT1, (22)]. The colors on transporters indicate the subfamilies of mitochondrial carriers, defined by substrate specificity; being red-orange for adenylates transporters; red for di-/tri-carboxylates transporters; dark-red for *S*-adenosylmethionine transporters; yellow for folate/FAD transporter; blue for amino acids transporters; orange for NAD transporters; gray for phosphate transporter; and green for thiamine pyrophosphate transporter. Abbreviations: Asp, aspartate; ANTs, adenine nucleotides; Basic AA, Basic amino acids; Cit, citrate; dNTPs, deoxynucleoside triphosphates; ER, endoplasmic reticulum; Glu, glutamate; IsoCit, isocitrate; SAM, *S*-adenosylmethionine; SAHC, *S*-adenosylhomocysteine; ThPP, thiamine pyrophosphate; ThMP, thiamine monophosphate.

Of the 58 MCF members in Arabidopsis, only 28 have thus far been reported to localize to the mitochondria by organellar proteomics and localization by fluorescent protein tagging, while a total of 12 MCF members have been reported (sometimes erroneously) to localize elsewhere [51,52]. As mentioned above, these lists are reliant either on the specific expression of fluorescent fusion proteins or are based on proteomics on highly purified organelles the results of which have been used to generate databases such as SUBA [53] and ARAMEMNON [54]. These studies reveal the presence of MCF members at several other locations including the peroxisome, plastid and endoplasmic reticulum (Figure 2) [29,35,55,56,57,58,59,60,61]. Here, we detail the localization experiments alongside characterization of mutants/transgenic lines of the transports focusing on the underlying mechanisms by which the in vivo function of these proteins are realized. Given that the function of those transporters whose function is intimately related to plant respiration have been reviewed very recently [52], we will only cover these in brief here and spend greater time discussing transporters with different functions.

### 3.1. Non-Mitochondrial MCFs

We include here all MCFs that have been reported as non-mitochondrial, although we stress that for at least one of these, highly convincing evidence exists that it is indeed mitochondrial. Three MCFs contain no N-terminal presequence and are localized at the endoplasmic reticulum (ER-ANT; [35]), Golgi apparatus (UCP2; [62]) or plasma membrane (PM-ANT; [36]). Current thinking suggests that these localizations are due to the specific transmembrane domain lengths in these proteins which resemble the average for their respective locations [63]. However, UCP2 has alternatively been reported to reside in the mitochondria [64] and as such its localization should currently be regarded as unclear. Six further MCF members have been reported to be found in plastids—with four of these proteins having cleavable N-terminal presequences and in the case of Brittle 1, the removal of this presequence targeted the protein to the mitochondrion [34]. One of the two transporters lacking a cleavable N-terminal presequence NDT1 was subsequently convincingly demonstrated not to be plastidic but rather mitochondrial by a wide range of evidence (see [27] for details). This casts doubt on localization results based purely on fluorescent marker proteins and acts as a cautionary note that it is better to adopt multiple strategies for assignment. As such, we will discuss the function of NDT1 in detail, below; however, given that some ambiguity exists concerning the location of UCP2 we will treat this as a non-mitochondrial member for now. The final members of this list (PNC1/2 and PXN) all reside in the peroxisome despite the fact that none of them contained either classical PTSI C-terminal sequence or the N-terminal presequences observed for plastid targeted MCF proteins [57,65].

Assigning the location of these proteins, as well as assessing their patterns of transcription, provides some contextualization for their in vivo function. However, far more insight can be achieved by evaluating the biological roles of the transporters by assessing plants deficient in the expression of the transporters in question and when possible comparative physiology between these mutants and their counterparts in yeast. Of the 11 family members (at least putatively) assigned a non-mitochondrial location; however, this is often not possible. That said the peroxisomal nucleotide carriers PNC1 and PNC2 are functionally highly similar to their yeast counterparts [57,66]. Investigation of transgenic Arabidopsis lines has revealed that PNC1 and PNC2 play an essential role in energy provision via their catalysis of the counter exchanges of ATP with ADP or AMP to plant peroxisomes, and that lack of these proteins impairs fatty acid breakdown and other peroxisomal reactions, including auxin metabolism [57]. The third reported MCF that localizes to the peroxisome is PXN, which was confirmed to catalyze NAD^+^ uptake in exchange mainly with AMP but also with NADH, ADP and nicotinate adenine dinucleotide. The absence of this carrier in Arabidopsis led to an accumulation of oil bodies in seedlings and an impaired peroxisome localized β-oxidation [65]. The ER-ANT1 Arabidopsis knockout mutant exhibits stunted growth but survives and produces fertile seeds [67], this work thus suggested the presence of a further carrier(s) capable of energy provision to the ER. The presence of such a transporter was recently supported by the characterization of the mammalian AXER protein—for which an Arabidopsis homolog has been identified [68]. By contrast, the PM-ANT1 is highly expressed in developing pollen and plants exhibiting a mutation in this carrier are impaired in flower development particularly during anther dehiscence. These phenotypes were interpreted to suggest that the transporter mediates ATP export specifically from pollen cells and that enhanced eATP acts as a signal that is received by the stromium cells of the anther [36]. As for the ER-ANT, it can be supposed that Arabidopsis contains other proteins that mediate ATP export at the plasma membrane since the effects of downregulating PM-ANT were confined to the flower [24], while the role of extracellular ATP is far more widespread [69]. Before turning to the plastid localized members of the MCF—one final other member needs to be discussed, UCP2. As we mentioned above, it is currently ambiguous where this is localized with it variously being reported to localize to the Golgi apparatus and the mitochondria, as detailed in our accompanying article [15]. UCP2 displays similar substrate specificities to UCP1 [64] and knockout of either transporter in Arabidopsis resulted in similar metabolic phenotypes consistent with both transporting organic acids. However, notably, the metabolic phenotype of *ucp1ucp2* double mutants under salt stress is more reminiscent of the *ucp1* than the *ucp2* single mutant, suggesting that UCP1 plays the dominant role under these conditions [64]. This fact notwithstanding, proteomics analyses have suggested, in contrast to the GFP fluorescence studies of Monne et al. [64], that UCP2 actually resides at the Golgi apparatus [62]. As such, further research is imperative to establish both the location and function of this isoform.

Having described those few transporters (potentially) localized to the peroxisome, endoplasmic reticulum, Golgi apparatus and plasma membrane we next turn to the five MCF members believed to localize to the plastid. It is perhaps unsurprising that the most similar organelle to the mitochondria harbors so many MCF members or the breadth of substrates that they cover being reported to transport adenylates, folates, FAD, *S*-adenosine methione (SAM) and iron. Taking these one by one, two transporters have been implicated in adenylate transport pANT1 and the thylakoid lumen transporter TAAC [70]. pANT1 has been demonstrated to locate either to the plastid [61] in the presence of its N-terminal extension, or to the mitochondria in its absence [34]. However, as yet, unlike its paralog Brittle1 in maize [71,72], mutants of the Arabidopsis gene remain to be characterized. This is not the case for the TAAC transporter, however, with loss of function mutants leading to the assumption that this transporter mediated ATP provision to the thylakoid lumen. As such, it was suggested to play a role in photoinhibition and photoprotection of photosystem II, as well as regulating the electrochemical proton gradient across the thylakoid membrane [73]. However, although these roles are not obviated by the later finding that the TAAC is also located at the plastid envelope [70], they do probably need critical reassessment particularly given that its transcript and protein abundances also suggest it to play a role in developing plastids that are essentially free of thylakoids [24]. Next in the list is FOLT1, which catalyzes the transfer of folate and its derivatives [60,74]. The absence of major phenotypical differences in the loss of function mutant was taken to suggest the presence of an additional plastidial folate transport system with the fact that the major facilitator family transporter has been demonstrated to transport FAD [75], perhaps being part of such a system. A highly similar member of the MCF, namely NDT1, was additionally proposed to have a plastidial localization on the basis of GFP fluorescence analysis alone [26]; however, more comprehensive evaluation including that of Arabidopsis knockout mutant revealed that NDT1, like NDT2, actually resides at the inner mitochondrial membrane (IMM) [27,28]. We discuss this in detail below. Suffice it to say the fact that plastids lack the enzymatic machinery to make NAD^+^ means that a transporter capable of importing it into the plastid remains to be found. More clear is the localization of the SAM and *S*-adenosylhomocysteine (SAHC) transporter. SAM, like NAD^+^, is synthesized exclusively in the cytosol and has to be exported to the organelles where it is needed as substrate [76]. For this purpose Arabidopsis harbors two homologs to the transporters from yeast and mammalia [30,77], the N-terminal sequence of SAMC1 targets it to the plastid whereas SAMC2 resides at both plastid and mitochondrial membranes [29,31]. Moreover, loss of SAMC1 function resulted in a dwarf phenotype and a compromised prenyl lipid metabolism [29]. Finally, the mitoferrin-like transporter Mfl1 is a component of the inner plastid envelop and investigations into the corresponding mutant plants suggest its involvement in iron uptake by the chloroplast and revealed that it displayed reduced vegetative growth [78].

### 3.2. Mitochondrial MCFs

Counterintuitively, the mitochondrially localized MCFs are arguably less well characterized than those that localize to other membrane systems—largely due to the complexities that arise as a result of overlap or even redundancy of function. That said, several of the 28 proteins which have been identified to be present at the IMM have been well characterized, and many more have been at least partially characterized. While we grouped the transporters described in the previous section on the basis of their location here it makes more sense to group according to substrate specificity.

#### 3.2.1. Coenzyme A Transporters

Plants produce coenzyme A (CoA) in the cytosol which is than imported into the organelles where it is used in essential pathways such as the tricarboxylic acid (TCA) cycle in the mitochondria, fatty acid synthesis in the chloroplast, and β-oxidation in peroxisomes. A plant peroxisomal CoA transporter also able to transport NAD was identified and named peroxisomal NAD^+^ carrier (PXN; [79]) and also discussed in the previous section. In addition to CoA, PXN was shown to be able to accept as substrates NAD^+^, NADH, AMP, ADP, and adenosine 3′,5′-phosphate [65,79]. The lack of PXN in Arabidopsis seedlings delays the breakdown of fatty acids released from storage oil and thereby leads to the retention of oil bodies. This phenotype indicates that a defective PXN function lead to defects in β-oxidation during seedling establishment suggesting that PXN delivers NAD^+^ for optimal fatty acid degradation during storage oil mobilization [65]. Regarding specifically the mitochondrial CoA transporters, MCFs members putative CoA transporters were first identified in *Saccharomyces cerevisiae* [80] and mammals [81]. Next, comparative genomic analysis showed that nonflowering plants have one homologs of these mitochondrial CoA transporters, whereas in angiosperms plants have two distinct homologs [82]. The homolog proteins from maize (GRMZM2G161299 and GRMZM2G420119) and Arabidopsis (At1g14560 and At4g26180) are able to complement the growth defect exhibited by yeast mitochondrial CoA carrier mutant and also restore its mitochondrial CoA level, suggesting that these proteins have CoA transport activity in mitochondrial membrane [82]. Despite current knowledge related to the identity of CoA transporter candidates and the important function of CoA for mitochondrial reactions in plants, functional characterization of mutant plants, as well as biochemical properties, such as substrate specificities, still remain to be investigated.

#### 3.2.2. Phosphate Transporters

The orthophosphate (Pi) uptake by the mitochondrial matrix is essential for the oxidative phosphorylation of ADP to ATP. In Arabidopsis, three genes were identified as encoding mitochondrial Pi carriers (*At*MPTs), all members of MCF [83]. Expression analysis demonstrated that *At*MPTs are upregulated by high-salinity stress in *A. thaliana* seedlings [84]. Overexpressing *At*MPTs in Arabidopsis resulted in plants with a higher sensitivity to salt stress during seed germination and seedling establishment stages, as well as higher ATP content and energy charge in comparison with wild-type plants under salt stress. Further analyses revealed that activity *At*MPTs might be involved with gibberellin metabolism in *A. thaliana* during salt stress. Recently, it was shown that *At*MPT3 overexpression displays multiple developmental defects in Arabidopsis plants including dwarfed stature and reduced fertility [85]. In addition to changes in transcription of genes involved in plant metabolism and leaf and flower development, AtMPT3 overexpressing plants exhibited higher ATP content, faster respiration rate, and increased reactive oxygen species (ROS) production. Taken together, these studies demonstrated the importance of MPTs activity for plant growth and development under optimal and adverse conditions, through complex regulatory mechanisms related not only with ATP production but also with development and signaling processes.

#### 3.2.3. NAD Transporters

In addition to the peroxisome NAD transporter (PXN) described above, two other MCF members, namely *At*NDT1 and *At*NDT2, are able to catalyze the import of NAD in organelles [26]. These proteins were able to complement the phenotype exhibited by yeast mutant lacking NAD^+^ transport [26]. In the same study, it was demonstrated that *At*NDT1 and *At*NDT2 are capable of importing NAD^+^ against ADP or AMP, and do not accept NADH, NADP^+^, NADPH, nicotinamide or nicotinic acid as transport substrates. Despite the similarities in terms of biochemical properties of these transporters, initial GFP-protein localization analysis indicated that *At*NDT1 was located in the plastid membrane and *At*NDT2 in the mitochondrial membrane [26]. However, as discussed in the previous section, the plastid localization of *At*NDT1 was, for a long time, not well accepted, since GFP-tagging and immunolocalization analyses were not able to find *At*NDT1 targeted to chloroplast membranes [60] and a recent proteome study identified *At*NDT1 in mitochondrial membranes [86]. Recently, both *At*NDT1– and *At*NDT2−GFP fusion proteins were found exclusively located in the mitochondria, clearly indicating their mitochondrial localization [27]. Despite the similar biochemical properties and the same subcellular localization, the biological characterization of *At*NDT1 and *At*NDT2 proteins revealed that both proteins play important and non-redundant functions in Arabidopsis plants [27,28]. Physiological and metabolic analyses of plants with reduced *At*NDT1 expression, revealed increased leaf number and leaf area which was concomitant with increased photosynthetic activity and starch accumulation [27]. In addition to other analyses, these results suggested that downregulation of *At*NDT1 alters NAD^+^ metabolism and transport, leading to metabolic shifts which increased photosynthesis, activation state of the stromal NADP dependent malate dehydrogenase (NADP-MDH) and starch accumulation. Moreover, it was verified that plants with impaired *At*NDT1 transport exhibited reduced pollen grain viability, tube growth, short siliques and higher rate of seed abortion, demonstrating the important role of *At*NDT1 in reproductive tissues. Similarly, plants with reduced expression of *At*NDT2 were affected in reproductive phase [28]. The plants with impaired NDT2 transport exhibited a reduced seed yield, followed by reduced seed germination and retardation in seedling establishment. Remarkably, NDT2 mutants exhibited changes on primary metabolism in dry and germinated seeds and an increase in fatty acid levels observed during seedling establishment. Interestingly, flowers and seedlings of NDT2 mutants displayed upregulation of *de novo* and salvage pathway genes encoding for NAD biosynthesis enzymes, suggesting that these genes have a transcriptional control mediated by NDT2 activity. Recently, it was suggested that *At*NDT2 protein might be a key regulator of the mitochondrial NAD^+^ and NADH pools and compromised NAD^+^ import activity in *ndt2* mutants cannot be fully compensated for by other transporters [87], highlighting the importance role of NDT2 for NAD^+^ import by plant mitochondria. Taken together, these results suggest that correct NDT1 and NDT2 expression is necessary for maintaining NAD^+^ balance among organelles that modulate metabolism, physiology and developmental processes in plant tissues.

#### 3.2.4. Uncoupling Proteins

Uncoupling proteins (UCPs) have been described as being involved in the dissipation of proton gradients across the IMM that is normally used for ATP synthesis [64,88]. Based on homology with UCP from humans, former studies identified six genes in Arabidopsis genome (*At*UCP1–6) encoding putative UCPs [89]. Formerly, it was shown that the isoform *At*UCP1 (At3g54110) is localized to IMM and exhibits the activity of uncoupling protein similar to the human UCP1 [88,89,90]. The function of the other isoform *At*UCP2 (At5g58970) was less understood because it was detected in the Golgi apparatus [62] and also in the plasma membrane [63]. Recently it was shown that, exactly like *At*UCP1, *At*UCP2 is also a mitochondrial localized protein [64]. Astonishingly, both *At*UCP1 and *At*UCP2 were shown to be able to transport amino acids (glutamate, aspartate, cysteine sulfinate, and cysteate), dicarboxylates (malate, oxaloacetate, and 2-oxoglutarate), phosphate, sulfate, and thiosulfate [64]. In addition, it was verified that both proteins catalyze an electroneutral aspartate/glutamate heteroexchange activity, in contrast to that mediated by the mammalian mitochondrial aspartate glutamate carrier. Three other former members of the *At*UCP subfamily of Arabidopsis MCF (*At*UCP4-6) were renamed as dicarboxylate carriers (*At*DIC1-3), because these proteins transport oxaloacetate, malate, succinate, phosphate, sulfate, thiosulfate, and sulfite [91].

Regarding the physiological role of UCP proteins in plants, several studies have been performed. In Arabidopsis plants, the silencing of *At*UCP1 resulted in lower photosynthetic rates, specifically caused by restricted photorespiration, with reduced oxidation of photorespiratory glycine in the mitochondrion [88]. This study indicated that the function of *At*UCP1 is related to maintaining the redox poise of the mitochondrial electron transport chain and thus facilitating the photosynthetic metabolism in the chloroplast [88]. Uncoupled mitochondrial respiration might be important in plants undergoing stress situations, during which both respiration and photosynthesis may be impaired. In agreement, overexpressing *At*UCP1 in the IMM increases uncoupling respiration, reducing the cellular ATP content, and also decreasing the accumulation of reactive oxygen species (ROS) under abiotic stresses [92]. Transcriptome and metabolite analyses demonstrated that UCP1 overexpression in tobacco plants induces a hypoxic stress that disrupts cellular energy homeostasis and triggers a reconfiguration of metabolism [93]. Under stress conditions, the UCP activity would maintain the redox poise inside the mitochondria and in the chloroplasts allowing photosynthesis and mitochondrial activity. To verify the role of UCP1 in plant responses to drought stress, it was hypothesized that UCP1 overexpression would help tobacco plants cope with drought stress [94]. As expected, the UCP1 overexpressing plants maintained higher rates of respiration and photosynthesis and reduced the levels of H_2_O_2_ in leaves during the drought stress period. Together, these results demonstrated the importance of UCP1 under both optimal conditions and drought stress [94]. These results clearly demonstrate the importance of UCP1 in plant stress responses.

As mentioned above, in addition to the uncoupling function of UCPs, it was recently demonstrated that *At*UCP1 and *At*UCP2 are able to transport of amino acids and dicarboxylic acids through the IMM [64]. It is also suggested that *At*UCP1 and *At*UCP2 also catalyze an aspartate _out_/glutamate _in_ exchange across the mitochondrial membrane and, thereby, contribute to the export of reducing equivalents from the mitochondria in photorespiration [64]. Notably, *At*UCP1 and *At*UCP2 have broad substrate specificities, especially the dicarboxylates intermediates of TCA cycle. Thus, in agreement with previously proposed role of *At*UCP1 in photorespiration and photosynthesis [88], the role of *At*UCP1 and *At*UCP2 might be related with glycolate pathway for the shuttling of redox equivalents across the mitochondria as part of the malate/aspartate shuttle [52,64].

#### 3.2.5. Organic Acid Transporters

Several metabolites associated with the activity of TCA cycle should be exchanged across the IMM to link several mitochondrial enzymes to those in other cellular compartments [51,95]. In plants, three sub classes of MCF members are involved in the transport of organic acids, which are likely relevant for the activity of TCA cycle and reactions occurring in other organelles: dicarboxylic acid carriers (DICs), dicarboxylic /tricarboxylic acid carriers (DTCs), and succinate/fumarate carrier (SFC). These transporters are discussed in the following subsections.

##### Dicarboxylic Acid Transporters

As indicated above, in the Arabidopsis genome, three potential homologues of yeast and mammalian mitochondrial dicarboxylate carriers (DICs), previously reported as *At*UCP4-6, were described and designated as *At*DIC1-3 (*At*DIC1, At2g22500; *At*DIC2, At4g24570; and *At*DIC3, At5g09470) [91]. *At*DIC3 shares only 55–60% identical amino acids with *At*DIC1 and *At*DIC2, whereas *At*DIC1 and *At*DIC2 share 70% identical amino acids, suggesting that *At*DIC1 and *At*DIC2 are more closely related [91]. In a recent mitochondrial proteomic study it was verified that *At*DIC3 is not as highly expressed as *At*DIC1-2, with *At*DIC1 being more abundant than *At*DIC2 (59 and 21 protein copies per mitochondria respectively) [96]. The Arabidopsis DICs transport several dicarboxylates including malate, oxaloacetate and succinate as well as phosphate, sulfate and thiosulfate at high rates, whereas 2-oxoglutarate was revealed to be less preferred substrate. The kinetic properties of recombinant *At*DIC1-3 proteins were also evaluated [91]. Despite the identification and characterization of the biochemical properties of DICs proteins in Arabidopsis, the physiological functions of these transporters have still not been elucidated. Surprisingly, according to our current knowledge, the isolation and characterization of mutant plants for each *At*DIC isoforms still need to be performed. This fact led us to different questions regarding the physiological roles of these carriers in plants under distinct physiological conditions.

##### Dicarboxylic/Tricarboxylic Acid Transporters

Dicarboxylate/Tricarboxylate carriers (DTCs) are proteins that catalyze the transport of dicarboxylic acids (such as malate, maleate, oxaloacetate and 2-oxoglutarate) and tricarboxylic acids (such as citrate, isocitrate, *cis*-aconitate and *trans*-aconitate) across the IMM [97]. These transporters are the most abundant MC proteins in the IMM of Arabidopsis, comprising about 0.8% of the total IMM area (6836 protein copies per mitochondria) [96]. Unlike the other three more abundant carrier proteins in the IMM (ADP/ATP carriers (*At*AAC1-3) and mitochondrial phosphate carriers (*At*MPT2-3) and uncoupling proteins (*At*UCP1-3), only one DTC homolog was identified in Arabidopsis (At5g19760). DTC proteins have also been reported in different plant species [97,98,99,100,101]. Interestingly, the number of DTC homologs in different plant species varies without a pattern; in the Brassica genus, the number of DTC homologs varies from one in *A. thaliana* and *Arabidopsis lyrata*, two in *Brassica oleracea*, and three in *Brassica rapa* [52], and four in tobacco (*Nt*DTC1-4) [97]. Biochemical characterization of *At*DTC and *Nt*DTCs revealed that the transport activity of these proteins involves an obligatory electroneutral exchange of dicarboxylates such as malate and 2-oxoglutarate and tricarboxylates such as citrate [97]. Furthermore, it was demonstrated that DTCs are able to catalyze homoexchange transport activities, such as dicarboxylate/dicarboxylate and tricarboxylate/tricarboxylate [97]. This biochemical characterization of DTCs also demonstrated that these proteins are able to transport several intermediates of the TCA cycle, with the exception of succinyl-CoA and fumarate, for which there is no available information.

##### Succinate/Fumarate Transporter

Considering that degradation of storage compounds at early stages of seedling development is essential to plant development, providing energy and intermediates required for construction of the photosynthetic apparatus and thus allowing autotrophic growth, the transport of metabolites from and into mitochondria is essential. In this regard, a homologue of the mitochondrial succinate/fumarate carrier from yeast (Sfc1p) was identified in the Arabidopsis genome and named as *At*SFC1 [102]. Recently, biochemical characterization of the *At*SFC1 encoded protein demonstrated that this carrier transports citrate, isocitrate and aconitate and, to a lesser extent, succinate and fumarate [103]. Further gene expression analysis in Arabidopsis indicated that *At*SFC1 is highly expressed in heterotrophic tissues. In agreement, lower expression of *At*SFC1 reduced seed germination and impaired radicle growth, a phenotype that was related with reduced root respiration rate. Together, these results suggested that *At*SFC1 is involved in storage oil mobilization at early stages of seedling growth and might be important for nitrogen assimilation in root tissues by catalyzing citrate/isocitrate or citrate/succinate exchanges [103]. Notwithstanding that SFC1 was previously supposed to be a succinate/fumarate carrier [102], the fact that mitochondria lack the transport machinery capable of importing succinate into the mitochondria from lipid mobilization during seed germination remains to be understood.

#### 3.2.6. Amino Acid Transporters

In plants, as well as in other organisms, mitochondria play an important role in amino acid metabolism. Several intermediates needed for amino acid biosynthesis are intermediates of the TCA cycle, and, conversely, amino acids may be converted into TCA cycle intermediates [104]. In addition, reactions involved in the catabolism of amino acids take place in mitochondria [48,105]. Thus, amino acid transporters must play important roles in the import of amino acids and the export of precursors for biosynthetic pathways.

Research efforts have been devoted to understanding the roles of a putative amino acid transporter named A BOUT DE SOUFFLE (BOU), which was identified in Arabidopsis (At5g46800) for a long time [106]. Physiological characterization of BOU transporter in plants indicated that this protein plays important roles in fatty acid β-oxidation [106], photorespiration and growth of meristem cells [107]. Seedlings from the *bou* mutant stopped developing after germination and degradation of storage lipids but were not able to proceed towards autotrophic growth. Further analyses revealed that the *bou* mutant’s post germination phenotype is similar to that displayed by mutants impaired in fatty acid β-oxidation indicating that BOU might be a mitochondrial acyl-carnitine carrier [106]. Further studies demonstrated that BOU gene is co-expressed with photorespiratory genes in leaf tissues, suggesting that this transporter might be involved with photorespiration [107]. Physiological characterization of the knockout mutant *bou-2* showed that the mutant plants exhibit the typical photorespiratory growth phenotype, together with elevated CO_2_ compensation point and glycine accumulation. Furthermore, it was observed that the shoot apical meristem organization is compromised in seedlings from the *bou-2* line. These results demonstrated that BOU transporter might be involved in photorespiratory metabolism and is necessary for meristem growth at ambient CO_2_ [107]. Despite the studies indicating the important function of BOU transporter in plants, the specific substrate for the BOU transporter protein was revealed only recently [108]. Detailed biochemical characterization of Arabidopsis BOU and YMC2P from *S. cerevisiae* revealed the transport properties and kinetic parameters of these proteins. Both YMC2P and BOU proteins are able to transport glutamate, but not other amino acids or many other tested metabolites [108]. Together these studies demonstrated that BOU protein, by importing glutamate into mitochondria, plays an important role in carbon and nitrogen metabolism and potentially also mitochondrial protein synthesis.

In Arabidopsis, another two MCF members, *At*BAC1 (At2g33820) and *At*BAC2 (At1g79900), catalyze the transport of basic amino acids through the IMM [9,109,110,111]. Sequence analysis indicated that *At*BAC1 shares 36% of identity with BOU, whereas *At*BAC2 is 40% similar to the human SLC25A29 transporter, although it is also related to BOU (36% identity) and aspartate/glutamate carriers (AGCs; 30–33% identity) [9]. Experiments with recombinant proteins from *At*BAC1 and *At*BAC2 reconstituted in liposomes indicated that both proteins transport lysine, arginine, ornithine and histidine [109,110]. These transporters exhibit differences in terms of substrate specificity; in comparison with *At*BAC1, the isoform *At*BAC2 is less specific for l-amino acids and also the only *At*BAC able to transport the neutral amino acid citrulline [109,110].

Regarding the physiological roles of these proteins, the two *At*BACs seem to play different functions in plants. It has been demonstrated that *At*BAC1 is likely involved in remobilization of storage compounds after seed germination in Arabidopsis and rice plants [109,111,112]. Meanwhile, *At*BAC2 seems to play an important role during stress recovery, since it seems to be more expressed in responses to hyperosmotic stress and also during dark induced senescence [39,40]. It was demonstrated that overexpression of *At*BAC2 in transgenic plants allows plants to use arginine as a source of nitrogen [39] and that this isoform of BAC is necessary for the complete recovery of leaf growth after hyperosmotic stress [40]. These results are in agreement with studies demonstrating that some amino acids accumulate in plant tissues during stress establishment and are degraded during the period of stress recovery [48,113]. Thus, the arginine transport, mediated by *At*BAC2, and degradation inside the mitochondria might be important in reducing the excess of arginine, recycling the nitrogen and urea and thus providing intermediates for the synthesis of primary molecules necessary for plant growth during stress recovery [40]. Furthermore, in the same study, transcription analysis revealed that under stress conditions *At*BAC2 expression affects the transcript levels of several genes such as those encoding stress-related transcription factors, arginine metabolism enzymes, and transporters. Taken together, these studies indicate the clear importance of basic amino acid mitochondrial transport in responses to hyperosmotic stress.

#### 3.2.7. Iron Transporters

Mitochondrial iron (Fe) transporters, also named Mitoferrins (mIT), were first identified and characterized in drosophila, zebrafish and humans [114,115,116]. In plants, a mIT homolog was first identified in rice [117]. In this species, a silenced mutant line for mIT resulted in a lethal phenotype. The mIT protein from rice was able to complement the growth of yeast mutant which was defective in mitochondrial Fe transport. Interestingly, the growth of mIT-knockdown rice mutant plants was impaired despite abundant Fe accumulation [117]. Further analyses of the rice mIT mutants revealed that Fe-s cluster synthesis is affected in the knockdown plants. These results clearly suggested that mIT plays an essential role for rice growth and development [117]. In Arabidopsis, two genes encode for mIT (*At*mIT1 and *At*mIT2) [118]. Both mITs from Arabidopsis belong to the MCF and exhibit homology with mITs from other organisms. Single *At*mITs mutant plants do not exhibit clear phenotypes, but in the double mutant plants, silenced for both genes showed embryo lethal phenotype were shown to be essential for Fe homeostasis and embryogenesis in Arabidopsis. Additional analyses demonstrated that both transporters are necessary for mitochondrial Fe uptake and also for the correct mitochondrial function. Together, these studies indicate that mITs are necessary for the maintenance of both mitochondrial and whole plant Fe homeostasis, and consequently essential for the proper growth and development of the plant.

## 4. Conclusions

Research into the in vivo functions of the plant mitochondrial carrier family has made impressive advances since the last comprehensive reviews were published some eight to nine years ago [14,23]. Indeed, despite the fact that lesser technological advances have been made than those described for the metabolic role of the transporters in the accompanying article [15], arguably greater progress has been made here. As we describe above, next-generation sequencing-based transcript profiling has greatly expanded the species and conditions for which expression analysis information is available for the plant MCF. Moreover, since 2012 a total of 21 MCF proteins have been characterized at the genetic level—largely by accession mutants of the various Arabidopsis T-DNA insertional mutant collections. Thus, we now have information on the effect of mutation in all of the major clades. That said a considerable number of gaps still need filling and even such well-studied proteins as the ATP/ ADP transporters have not been properly characterized in vivo. It seems likely that, due to functional redundancies, a range of double and triple mutants may be required in order to provide clearer clues in this direction. An additional area of interest for future work will be in elucidating the means by which these transporters are regulated in vivo. A wide range of post-translational modifications have been reported for plants [119], and many of these also occur within the MCF family; however, their physiological relevance is currently unclear. Despite these open questions immense advances have made within the last eight years and our understanding of plant organellar transport has been particularly enriched within this period.

## Supplementary Materials

The following are available online at https://www.mdpi.com/2218-273X/10/9/1226/s1, File S1.
